# Serotonin excites fast-spiking interneurons in the striatum

**DOI:** 10.1111/j.1460-9568.2009.06725.x

**Published:** 2009-04

**Authors:** Craig P Blomeley, Enrico Bracci

**Affiliations:** University of Manchester, Faculty of Life Sciences, AV Hill BuildingOxford Road, Manchester M13 9PT, UK

**Keywords:** 5-hydroxytryptamine, basal ganglia, GABAergic interneuron, rat

## Abstract

Fast-spiking interneurons (FSIs) control the output of the striatum by mediating feed-forward GABAergic inhibition of projection neurons. Their neuromodulation can therefore critically affect the operation of the basal ganglia. We studied the effects of 5-hydroxytryptamine (5-HT, serotonin), a neurotransmitter released in the striatum by fibres originating in the raphe nuclei, on FSIs recorded with whole-cell techniques in rat brain slices. Bath application of serotonin (30 μm) elicited slow, reversible depolarizations (9 ± 3 mV) in 37/46 FSIs. Similar effects were observed using conventional whole-cell and gramicidin perforated-patch techniques. The serotonin effects persisted in the presence of tetrodotoxin and were mediated by 5-HT_2C_ receptors, as they were reversed by the 5-HT_2_ receptor antagonist ketanserin and by the selective 5-HT_2C_ receptor antagonist RS 102221. Serotonin-induced depolarizations were not accompanied by a significant change in FSI input resistance. Serotonin caused the appearance of spontaneous firing in a minority (5/35) of responsive FSIs, whereas it strongly increased FSI excitability in each of the remaining responsive FSIs, significantly decreasing the latency of the first spike evoked by a current step and increasing spike frequency. Voltage-clamp experiments revealed that serotonin suppressed a current that reversed around −100 mV and displayed a marked inward rectification, a finding that explains the lack of effects of serotonin on input resistance. Consistently, the effects of serotonin were completely occluded by low concentrations of extracellular barium, which selectively blocks Kir2 channels. We concluded that the excitatory effects of serotonin on FSIs were mediated by 5-HT_2C_ receptors and involved suppression of an inwardly rectifying K^+^ current.

## Introduction

The striatum is the main input area of the basal ganglia and it plays a crucial role in motor control and reward-mediated learning (Bolam *et al.*, 2000; Bevan *et al.*, 2002; Graybiel, 2005; McHaffie *et al.*, 2005; Redgrave & Gurney, 2006). Striatal projection neurons, which receive strong glutamatergic inputs from the cortex and thalamus (Cherubini *et al.*, 1988; Mallet *et al.*, 2006), constitute > 95% of the striatal neuronal population (Graveland & DiFiglia, 1985). Despite their relative scarcity, however, GABAergic and cholinergic interneurons can control large striatal areas through their axonal arborizations and have emerged as a major target for neuromodulation (Kita *et al.*, 1990; Kubota & Kawaguchi, 2000; Tepper & Bolam, 2004). Among the GABAergic interneurons, fast-spiking interneurons (FSIs) are the most numerous class (Tepper & Bolam, 2004) and their ability to powerfully inhibit projection neurons has been demonstrated both *in vitro* (Koos & Tepper, 1999; Koos *et al.*, 2004) and *in vivo* (Mallet *et al.*, 2005, 2006). This inhibition is referred to as feed-forward because the FSIs receive a strong excitatory input from the cortex, which is also the major source of excitation for the projection neurons (Ramanathan *et al.*, 2002). The rarity of the FSIs and the fact that, unlike large cholinergic interneurons, they cannot be visually identified in brain slices, make systematic electrophysiological studies on these cells challenging. Nevertheless, we and other groups have shown that FSIs are excited by dopamine and acetylcholine (Bracci *et al.*, 2002; Koos & Tepper, 2002; Centonze *et al.*, 2003). Another neurotransmitter that is crucially involved in cognitive and motor functions is serotonin (Sandyk & Fisher, 1988; Meneses, 1999; Geyer & Vollenweider, 2008). This amine, whose function is characterized by a complex interaction with the dopaminergic system (Di Giovanni *et al.*, 2008), is released in the striatum by dense terminal projections mainly arising from the dorsal raphe nucleus (Lavoie & Parent, 1990; Vertes, 1991). Consistently, serotonin and its receptors are extremely abundant in the striatum (for a recent review see Di Matteo *et al.*, 2008). *In-vivo* experiments have shown that activation of serotonin receptors in the striatum elicits predominantly inhibitory responses in projection neurons (el Mansari *et al.*, 1994; el Mansari & Blier, 1997). The cellular mechanisms underlying these effects are not completely understood. We have previously shown that serotonin excites striatal cholinergic interneurons (Blomeley & Bracci, 2005) and that these cells, in turn, inhibit the glutamatergic input to projection neurons (Pakhotin & Bracci, 2007). In order to gain a more complete picture of the action of serotonin in the striatum, we have investigated the effects of this neurotransmitter on FSIs, which also exert a major inhibitory influence on the striatal output.

## Materials and methods

Wistar rats (both sexes, postnatal day 16–27, supplied by Biological Services Facility, University of Manchester, UK) were used for the experiments. Overall, 57 animals were used; the number of animals used for each experiment corresponds to *n* indicated in the Results, as each FSI was recorded from a different animal. The animals were killed using cervical dislocation, a humane method of killing in accordance with the UK Animals (Scientific Procedures) Act 1986 and the European Communities Council Directive (86/609/EEC). Coronal brain slices (300 μm thick) were obtained using a vibroslicer and maintained at 25°C in oxygenated artificial cerebrospinal fluid [composition (in mm): 126 NaCl, 2.5 KCl, 1.3 MgCl_2_, 1.2 NaH_2_PO_4_, 2.4 CaCl_2_, 10 glucose, 18 NaHCO_3_]. For recordings, slices were submerged, superfused (2–3 mL/min) at 25°C and visualized with infrared/differential interference contrast microscopy. Drugs were bath-applied. Current-clamp recordings were performed in bridge mode using an Axoclamp-2B amplifier (Axon Instruments) or a BA-1S bridge amplifier (NPI electronic GmbH). Voltage-clamp recordings were performed using the AxoClamp-2B in continuous single-electrode mode, with uncompensated series resistance. Whole-cell recordings were obtained with patch pipettes (2–5 MΩ) filled with a solution containing (in mm): 125 potassium gluconate, 10 NaCl, 1 CaCl_2_, 2 MgCl_2_, 1 BAPTA, 19 HEPES, 0.4 Mg-GTP, 4 Mg-ATP, and adjusted to pH 7.3 with KOH.

Input resistance was measured in current-clamp experiments by applying small negative current steps (0.5–1 s long), eliciting 2–10 mV deflections in FSIs. If the FSI was depolarized as a result of a pharmacological treatment, it was manually repolarized to control level for a short period while the current steps were applied; in this way, input resistance measurements were obtained at the same membrane potential. The input resistance was calculated by dividing the steady-state voltage deflection (measured at the end of the step) by the amplitude of the current step. For each pharmacological condition tested, 5–25 current steps were applied at 10 s intervals; the input resistance measurements for each condition were then grouped for statistical analysis.

For perforated-patch recordings, gramicidin (10–20 μg/mL) was added to the intrapipette solution and perforation was monitored as described in Blomeley & Bracci (2005).

The curve of best fit for the average current suppressed by serotonin was obtained using the equation: *I* = *g*(*V*_m_) × (*V*_m_ − *E*_k_), where *g*(*V*_m_) was described by a Boltzmann’s equation: *g*(*V*_m_) = *g*_max_((1 + exp(*V*_m_ − *E*_k_ − Δ*V*_h_))ν^−1^)^−1^. The parameters *g*_max_, Δ*V*_h_ and ν yielding the curve of best fit were obtained using the method of non-linear least squares provided by matlab software (The MathWorks). The slope conductance associated with the serotonin-suppressed current was estimated using the curve of best fit for *I*(*V*_m_); the voltage domain was divided into 1 mV intervals and the slope conductance was calculated as the ratio Δ*I* : Δ*V* for each of these intervals.

For action potential half-width measurements, the spike threshold was defined as the point where the rate of depolarization exceeded 75 mV/ms; spike amplitude was measured as the difference between the threshold level and the peak of the spike. For each FSI, 10–30 suprathreshold depolarizing current steps were applied every 10 s in each pharmacological condition, and the half-width of the first spike induced by each step was measured and used for statistical comparison.

Experimental values are expressed as mean ± SD and all statistical comparisons were carried out using non-directional Mann–Whitney *U-*tests (originpro 8 software). Drugs were obtained from Tocris Bioscience UK, apart from 5-hydroxytryptamine (5-HT) hydrochloride and gramicidin, which were obtained from Sigma-Aldrich UK.

## Results

### Electrophysiological identification of striatal fast-spiking interneurons

**Fig. 1:**
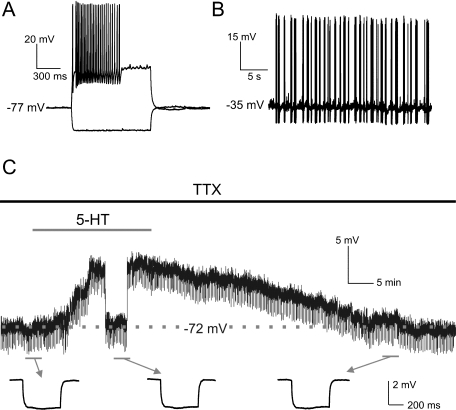
Effects of serotonin on FSIs in the presence of TTX. (A) Electrophysiological properties of an FSI revealed by current injections (± 100 pA). (B) Rhythmic intermittent firing was observed in another FSI depolarized by a steady current injection. (C) Serotonin (30 μm) reversibly depolarized an FSI in the presence of TTX (1 μm). Input resistance was monitored using current pulses (15 pA, 1 s) applied every 10 s. During the depolarization induced by serotonin, the FSI was manually repolarized to control level, to measure input resistance. Expanded traces are averages of 20 voltage deflections elicited by consecutive current pulses in each condition (control, serotonin and washout). No significant changes in input resistance were observed.

The first two postnatal weeks are critical for FSI development (Chesselet *et al.*, 2007). Thus, we used animals of postnatal day 16–27. We recorded from 49 striatal FSIs using conventional whole-cell techniques and from eight FSIs using gramicidin perforated-patch techniques (Blomeley & Bracci, 2005). FSIs were identified based on their distinctive electrophysiological features, including: (i) intermittent firing at high frequency; (ii) narrow action potentials (half-width < 1 ms); (iii) large spike afterhyperpolarizations; and (iv) subthreshold oscillations (amplitude 1–5 mV) observed between spike bursts (Kawaguchi, 1993; Bracci *et al.*, 2003). Examples of these properties are shown in Fig. 1A and B. The average resting membrane potential of the FSIs (in the absence of any injected current) was −69 ± 5 mV and the average input resistance was 203 ± 34 MΩ (average input conductance was 5.7 ± 1.9 nS). Access resistance (regularly compensated in bridge mode) was 3–4 MΩ and did not change by more than 30% in these experiments.

### Effects of serotonin on fast-spiking interneurons and receptors involved

In order to establish whether serotonin caused direct effects on FSIs, we carried out a series of experiments in the presence of the sodium channel blocker tetrodotoxin (TTX) (1 μm). Serotonin (30 μm, a dose that produces maximal effects in cholinergic interneurons) induced reversible depolarizing effects (8.4 ± 3.6 mV) in 4/5 FSIs tested in the presence of TTX using conventional whole-cell recordings (Fig. 1C). These depolarizations reached maximal value at 11.7 ± 1.5 min after the onset of serotonin application. In 4/4 cases in which a depolarization was observed, no significant changes in the apparent cell input resistance were observed during serotonin application (as shown in the example of Fig. 1C).

**Fig. 2:**
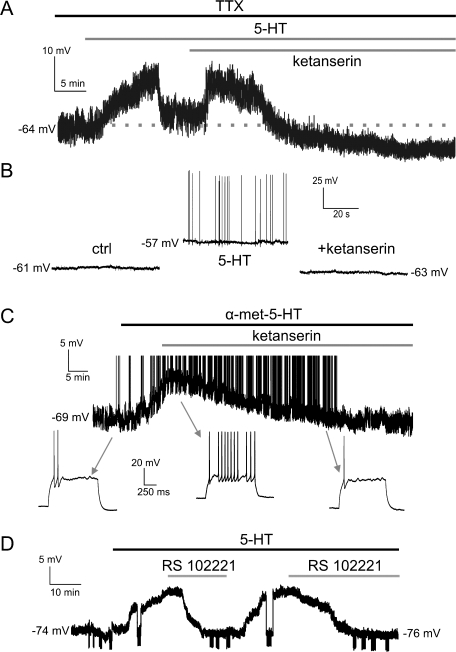
Serotonin effects are mediated by 5-HT_2_ receptors. (A) Serotonin depolarized an FSI in the presence of TTX (1 μm). Subsequent addition of ketanserin (10 μm) caused the FSI to hyperpolarize to a level more negative than control. During the depolarization induced by serotonin, the FSI was transiently repolarized to control level to measure input resistance (not significantly different from control). (B) In this experiment, an FSI (recorded without steady current injection) did not display any spontaneous firing activity in control solution. Serotonin induced a depolarization accompanied by spontaneous bursts of action potentials. Subsequent application of the 5-HT_2_ receptor antagonist ketanserin (10 μm) repolarized the FSI and abolished spontaneous firing. (C) Another FSI, with a resting membrane potential of −69 mV, was depolarized (∼9 mV) by the 5-HT_2_ receptor agonist α-methyl-5-HT (30 μm). Subsequent addition of ketanserin reversed these effects. All vertical deflections in the slow time-scale trace are depolarizations induced by current steps (50 pA, 1 s), which were delivered to monitor the excitability of the FSI. Three representative responses to such current steps in different pharmacological conditions are shown below the slow time-scale trace. (D) In this experiment, bath application of serotonin caused a depolarization (∼12 mV) from a resting membrane potential of −74 mV in an FSI; in the presence of serotonin, application of the selective 5-HT_2C_ receptor antagonist RS 102221 caused the membrane to repolarize to levels slightly more negative (−76 mV) than that observed in control solution. Washout of RS 102221 (still in the presence of serotonin) caused the membrane potential to depolarize again to a level similar to that observed in the presence of serotonin before RS 102221 application; subsequent reapplication of RS 102221 (still in the presence of serotonin) caused the FSI to repolarize again at −76 mV.

In another series of experiments, we applied serotonin in the absence of TTX to investigate its effects on FSI excitability. Under these conditions, serotonin induced reversible membrane depolarizations (8.9 ± 2.8 mV) in 27/33 FSIs recorded with conventional whole-cell techniques (time to maximal depolarization was 10.4 ± 3.0 min). In the 6/33 cells that did not respond to 30 μm serotonin, subsequent application of higher concentrations (60–120 μm) failed to elicit significant effects. In most cases, serotonin-induced depolarizations *per se* did not elicit action potentials. However, in five FSIs serotonin did cause the appearance of spontaneous action potentials (at an average frequency of 3.6 ± 2.1 Hz), as shown in the examples of Figs 2B and 6A. The input resistance was monitored in 16 FSIs depolarized by serotonin in control solution. As observed in the presence of TTX, we found that overall there were no significant changes in the apparent input resistance of these FSIs in the presence of serotonin. On average, the FSI input resistance measured with small current steps in the presence of serotonin was 96 ± 5% of that observed in control solution.

In other basal ganglia neurons, serotonin depolarizing effects are mediated by 5-HT_2_ receptors (Blomeley & Bracci, 2005; Stanford *et al.*, 2005; Bonsi *et al.*, 2007). To test the involvement of these receptors, we applied the 5-HT_2_ receptor antagonist ketanserin (10 μm) in nine experiments in which serotonin had depolarized FSIs by > 5 mV (four of these experiments were carried out in the presence of TTX). In 9/9 cases, ketanserin (applied in the presence of serotonin) fully reversed the serotonin effects, causing the membrane potential to return either to the level observed in control solution (5/9 cells, two of which were recorded in the presence of TTX) or to more hyperpolarized levels (on average by 3.1 ± 1.4 mV; 4/9 cells, two of which were recorded in the presence of TTX). Two cases in which ketanserin caused the membrane potential to attain levels more negative than in control solution are presented in Fig. 2A and B.

To further test the hypothesis that the effects of serotonin were mediated by 5-HT_2_ receptors, we also investigated the effects of the 5-HT_2_ receptor agonist α-methyl-5-HT (30 μm) on FSIs. α-methyl-5-HT produced depolarizing effects (8.0 ± 1.7 mV) similar to those of serotonin in 6/7 FSIs, as shown in the example of Fig. 2C. In 6/6 FSIs depolarized by α-methyl-5-HT, the subsequent addition of ketanserin completely reversed these effects (not shown).

These data clearly indicated that serotonin depolarized FSIs acting on 5-HT_2_ receptors. The 5-HT_2A_ and 5-HT_2C_ receptor subtypes are abundantly expressed in the striatum (Di Matteo *et al.*, 2008); 5-HT_2A_ receptors are mainly found in medium spiny projection neurons (Rodriguez *et al.*, 1999). We therefore investigated whether the effects of serotonin on FSIs were mediated by 5-HT_2C_ receptors, using the subtype-specific antagonist RS 102221 (Stanford *et al.*, 2005). In five FSIs in which serotonin had caused a depolarization (9.7 ± 2.2 mV), subsequent application of RS 102221 (1 μm), still in the presence of serotonin, fully reversed these effects, causing the membrane potential to return to levels slightly more negative (on average by 1.1 ± 1.4 mV) than that observed in control solution. In four of these five FSIs, RS 102221 was then washed out, still in the presence of serotonin, and subsequently reapplied (after > 10 min). In all cases, removal of RS 102221 caused the FSI membrane potential to return to depolarized levels similar to those observed during the first application of serotonin, whereas reapplication of RS 102221 caused repolarizations to levels slightly more negative than that observed in control solution. An example of these effects is illustrated in Fig. 2D; in this experiment, RS 102221 was applied and washed out twice in the presence of serotonin. The ability of RS 102221 to completely reverse the effects of serotonin showed that the effects of serotonin on FSIs were entirely mediated by 5-HT_2C_ receptors.

In control solution, the effects of serotonin or α-methyl-5-HT on FSI excitability were tested by repeatedly depolarizing these cells with current injections before and after drug application. An example of these experiments is illustrated in Fig. 2C. In this case, the current steps (50 pA, 1 s) elicited 2 ± 1 spikes in control solution, whereas the same steps elicited 18 ± 3 spikes in the presence of α-methyl-5-HT (*P* < 0.001). A Mann–Whitney *U* test was carried out on the number of spikes elicited by 10 consecutive steps in control solution and 10 in the presence of α-methyl-5-HT; in each case the steps were applied every 10 s; when ketanserin was added, the number of spikes decreased back to 2 ± 1 (10 steps). Similar results were observed in 6/6 FSIs, in which either serotonin or α-methyl-5-HT had induced, *per se*, subthreshold depolarizations; in all cases current steps elicited significantly more spikes in the presence of serotonin or α-methyl-5-HT than in control solution (*P* < 0.001; calculated on number of spikes elicited by at least 10 steps in each experiment for each pharmacological condition).

**Fig. 3:**
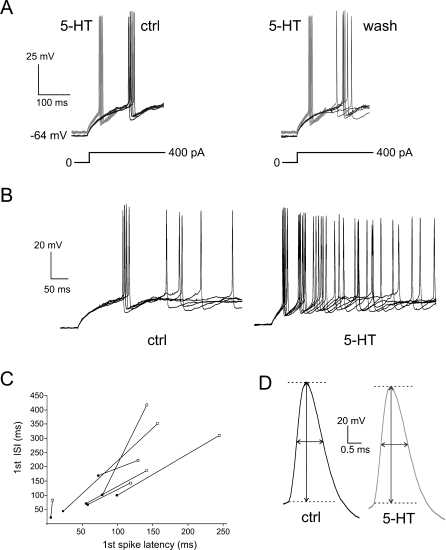
Effects of serotonin on FSI excitability. (A) In a representative FSI, suprathreshold depolarizing current pulses were applied in control solution, in the presence of serotonin (which had caused a 4 mV depolarization) and after serotonin washout. Five consecutive traces (interval 10 s) are superimposed for each condition. Serotonin caused a large decrease in the latency of the first spike and these effects were reversed on washout. Black traces were recorded in control solution, before (left panel) and after (right panel) serotonin application; grey traces were recorded in the presence of serotonin and are truncated earlier to avoid overlapping of the second spike with the first spike of the traces recorded in control solution. (B) These traces, taken from the same experiment as in A, show that in the presence of serotonin the first inter-spike interval (ISI) was strongly reduced; it can also be appreciated that the temporal scattering of the second spike evoked by a current step is greatly reduced in the presence of serotonin, resulting in a much lower variability of the first ISI. (C) Average results for seven FSIs to which the protocol illustrated in A and B was applied; the average first ISI and the average latency of the first spike for each FSI are represented by a point in a scatter plot in control solution (open circle) and another point in serotonin (black circle). The points in the two conditions for each FSI are connected by a line. A strong decrease in both parameters in the presence of serotonin is apparent for each cell. (D) The half-width of the action potentials evoked by current steps in control solution and in the presence of serotonin did not differ significantly. Average of the first spikes elicited by 10 consecutive steps (delivered at 10 s intervals) for each condition. Dashed lines indicate the threshold and peak of each action potential; double arrowed vertical bars represent the action potential amplitude; double arrowed horizontal bars represent the action potential half-width.

In another series of experiments, we used current steps that elicited at least two spikes in control solution. At least 10 identical steps were applied (every 10 s), in control solution and in the presence of serotonin, to seven FSIs, which were recorded without steady current injections. In control solution, the latency of the first spike was 134 ± 35 ms and the first inter-spike interval was 245 ± 88 ms. In each of these seven FSIs, serotonin induced depolarizations of > 5 mV and significantly (*P* < 0.01; 10–20 measurements per cell per condition) decreased the latency of the first spike (on average by 53 ± 10%) and the first inter-spike intervals (*P* < 0.03; 10–20 measurements per cell per condition) (on average by 63 ± 8%). An example of the effects of serotonin on spike latency, and their reversal after serotonin washout, is shown in Fig. 3A. An example of the effects of serotonin on the first inter-spike interval is illustrated in Fig. 3B. The average changes induced by serotonin on spike latency and the first inter-spike interval for each neuron tested are illustrated in Fig. 3C. Collectively, these experiments clearly showed that, even when the depolarizing effects of serotonin or α-methyl-5-HT were *per se* subthreshold, they strongly facilitated spike generation induced by concomitant excitatory stimuli.

In a previous study on cholinergic interneurons, the effects of serotonin could only be observed using perforated-patch techniques (Blomeley & Bracci, 2005). The observations reported so far with conventional whole-cell recordings show that this was not the case for FSIs; this is consistent with the observation that the physiological properties of FSI, unlike cholinergic interneurons, do not run down during long (> 2 h) conventional whole-cell recordings. Nevertheless, we carried out some perforated-patch recordings to test whether serotonin elicited additional effects not observed with conventional recordings. Under these conditions, in control solution, serotonin depolarized 6/8 FSIs and elicited no effects in 2/8 cells. The maximal amplitude of these depolarizations (8.1 ± 2.2 mV) and the time to peak (10.9 ± 2.8 min) were not significantly different from those observed with conventional whole-cell recordings (*P* = 0.94; *n* = 6 for gramicidin-perforated experiments and *n* = 27 for conventional whole-cell recordings). We concluded that the use of perforated patch was not critical to study serotonin effects on FSIs.

### Conductances modulated by serotonin

**Fig. 4:**
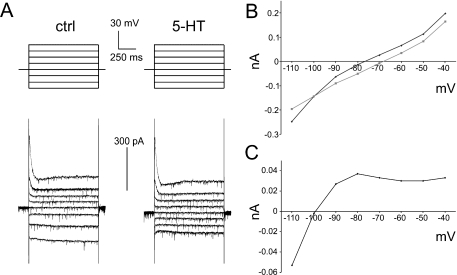
Membrane conductances modulated by serotonin revealed by voltage-clamp experiments. (A) A representative example of the voltage-clamp experiments carried out in the presence of TTX. Voltage steps (1 s) were applied to an FSI from the holding value (−80 mV) to levels between −100 and −40 mV (in 10 mV increments), in control solution and in the presence of serotonin. (B) Steady-state currents (recorded at the end of each voltage step) plotted vs. voltage in control solution (black) and in the presence of serotonin (grey) for the FSI of A. (C) Voltage dependence of the serotonin-suppressed currents in the same FSI. The steady-state current induced by serotonin was calculated, for each voltage, by subtracting the membrane current measured in control solution from that measured in the presence of serotonin. Serotonin-suppressed currents reversed around −100 mV and displayed prominent inward rectification.

The observation that the effects of serotonin persisted in the presence of TTX suggests that its action did not involve the voltage-activated sodium currents responsible for spike generation. Consistent with this notion, serotonin did not significantly (*P* > 0.07; at least 10 spikes per condition) affect action potential half-width in any of the 22 FSIs tested, as shown in the example of Fig. 3D. Although the action potential depolarizing phase is largely determined by sodium currents, Kv3.1–Kv3.2 potassium channels are mainly responsible for the repolarizing phase in FSIs (Erisir *et al.*, 1999). The absence of significant effects on action potential half-width also suggests that serotonin did not affect these conductances. We then investigated the action of serotonin on the currents active in the subthreshold membrane potential range. In order to do this, we carried out voltage-clamp experiments in seven FSIs in the presence of TTX. At *V*_h_ = −80 mV, serotonin induced an inward current in each of these seven FSIs (on average, −41.0 ± 5.7 pA). The time-course of serotonin responses was similar to that observed in current-clamp recordings. In four FSIs, a series of voltage steps (1 s long) were applied in control solution and in the presence of serotonin (after > 30 min from the onset of the application). These steps were delivered from a holding potential of −80 mV to levels between −110 and −40 mV, in 10 mV increments. A representative example of these experiments is shown in Fig. 4A. The steady-state current values for this FSI are plotted against membrane potential in Fig. 4B. The difference between steady-state currents in control solution and in the presence of serotonin is plotted as a function of voltage in Fig. 4C. This difference current, which represents the current suppressed by serotonin, reversed polarity at −98 mV and displayed a prominent inward rectification. Similar results were observed in the other FSIs. On average, the reversal potential of serotonin-induced current was −99.6 ± 1.0 mV, which is close to the nominal *E*_k_ (−104.3 mV). In order to quantify the inward rectification, we measured the slope conductance of the serotonin-suppressed current at −105 and −65 mV (see Materials and methods). This slope was significantly (*P* < 0.05, *n* = 4) larger at −105 mV (on average 5.3 ± 0.2 pA/mV) than at −65 mV (on average −0.4 ± 0.1 pA/mV), indicating the presence of strong inward rectification.

**Fig. 5:**
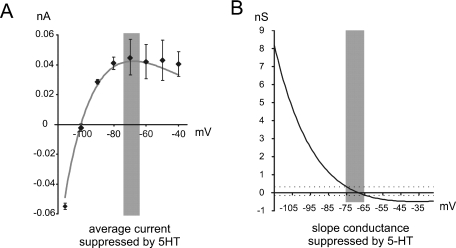
Serotonin-suppressed current has small slope conductance at FSI resting potential. (A) Diamonds represent the average steady-state current suppressed by serotonin at different voltages for four FSIs in which the protocol illustrated in A was carried out (error bars represent SEs). Superimposed (in grey) is a curve of best fit for these points (see Materials and methods for details). The area shaded in grey is centred around the average resting membrane potential of FSIs and spans two SDs (−69 ± 5 mV). (B) Estimated slope conductance associated with the serotonin-suppressed current, as a function of voltage. The curve of best fit shown in A was used to obtain this slope conductance. As in A, the area shaded in grey is centred around the average resting membrane potential of FSIs and spans two SDs (−69 ± 5 mV). The two horizontal dashed lines enclose the levels (between −0.15 and 0.25 nS) attained by the average slope conductance within this voltage range, where most resistance measurements were carried out.

The average current suppressed by serotonin for four FSIs is shown in Fig. 5A. A curve of best fit (see Materials and methods) for this average current is also superimposed in Fig. 5A. The slope conductance associated with the average serotonin-suppressed current was estimated using this curve (see Materials and methods) and is plotted in Fig. 5B. Such slope conductance is positive for voltages more negative than −68 mV but becomes negative for voltages more positive than −68 mV. This function represents an estimate of the contribution of the conductance suppressed by serotonin to the total slope conductance of the cell. The addition of a current with negative slope conductance produces a paradoxical decrease in the apparent conductance measured with small current injections in the corresponding voltage region (Westbrook & Mayer, 1984). The voltage region including −69 ± 5 mV (corresponding to the average FSI resting membrane potential ± SD) is highlighted in grey in both graphs. This is the voltage range in which the input resistance was measured both in control solution and in the presence of serotonin (when FSIs were manually repolarized with current injections). In this voltage region, the slope of the serotonin-suppressed current is effectively close to zero; as a result, the slope conductance of the serotonin-suppressed current is small, between −0.23 and 0.30 nS and vanishing at −68 mV, close to the average FSI resting potential. This observation therefore provided a biophysical explanation for the lack of statistically significant effects of serotonin on FSI input resistance observed in most cases (see Discussion).

**Fig. 6:**
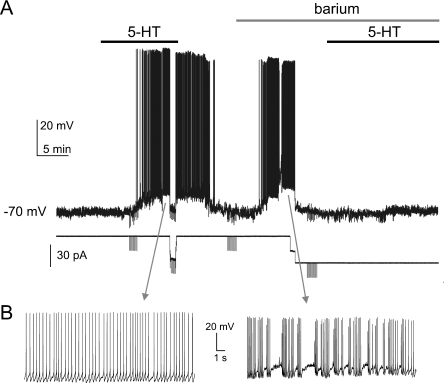
Barium (100 μm) occludes the excitatory effects of serotonin. (A) In this FSI, application of serotonin caused a reversible 10 mV depolarization, from a resting membrane potential of −70 mV; this depolarization was accompanied by spontaneous action potentials. After washout of serotonin effects, application of barium (100 μm) caused a depolarization (∼ 18 mV), also accompanied by spontaneous action potentials. In the continuous presence of barium, the FSI was manually repolarized to −70 mV (as indicated by the bottom trace depicting the injected current); under these conditions, subsequent application of serotonin failed to affect the FSI membrane potential. (B) Expanded traces (from the experiment in A) showing the spontaneous firing activity observed in the presence of serotonin (left) and barium (right).

Inwardly rectifying currents mediated by Kir2 channels are selectively blocked by low concentrations (100 μm) of extracellular barium (Chatelain *et al.*, 2005; Wilson, 2005). To test the involvement of these currents in the effects of serotonin, we used bath applications of barium chloride (100 μm) in five experiments in which a previous application of serotonin had caused depolarizing effects (8.6 ± 1.8 mV) in FSIs. Barium was applied after serotonin effects had completely washed out. Barium *per se* induced membrane depolarizations (12.0 ± 4.8 mV) in 5/5 FSIs. The input resistance was not significantly affected by barium in 5/5 cases (*P* > 0.05; at least five pulses for each condition). In the continuous presence of barium, the FSIs were manually repolarized at control level; under these conditions, reapplication of serotonin failed to affect the FSI membrane potential in all five cases. A representative example of these experiments is illustrated in Fig. 6. It should be noted that, in the absence of barium, reapplication of serotonin after complete washout of the effects of a first application elicited depolarizing effects similar to those of the first application (*n* = 4, not shown); thus, FSIs fully retained their ability to respond to serotonin after a first application.

We concluded that the effects of serotonin were completely occluded by the presence of extracellular barium; this observation strongly supports the conclusion that serotonin effects were mediated by a reduction in barium-sensitive inward rectifying potassium currents.

## Discussion

The main finding of this study is that serotonin directly depolarizes striatal FSIs, strongly increasing their excitability. This effect is mediated by activation of 5-HT_2C_ receptors and involves the suppression of an inwardly rectifying K^+^ current sensitive to low barium concentrations.

Our pharmacological experiments clearly identified 5-HT_2C_ receptors as responsible for serotonin effects on FSIs. In fact, the depolarizations induced by serotonin were completely reversed by ketanserin, a 5-HT_2_ receptor antagonist, and by RS 102221, which selectively blocks 5-HT_2C_ receptors. After the addition of ketanserin or RS 102221, the membrane potential often became more hyperpolarized than in control solution; this suggests that a tonic activation of 5-HT_2C_ receptors by ambient serotonin was present in control solution. In other brain regions, activation of 5-HT_2_ receptors causes excitatory effects in various neuronal types, although inhibitory effects have also been observed (Liu *et al.*, 2003; Stanford *et al.*, 2005).

Our voltage-clamp experiments allowed us to identify the biophysical properties of a conductance modulated by serotonin in FSIs. These data showed that serotonin suppressed a current that reversed very close to potassium reversal potential and displayed prominent inward rectification. Inward rectification is present in striatal FSIs (Kawaguchi, 1993) and Kir2 inwardly rectifying potassium currents are also prominent in striatal projection neurons (Nisenbaum & Wilson, 1995; Shen *et al.*, 2008). The observation that serotonin effects were completely occluded by barium, at a concentration that selectively blocks the Kir2 channels (Choe *et al.*, 1998; Wilson, 2005), demonstrated that excitation of FSIs was mediated by suppression of inwardly rectifying currents mediated by these channels. Interestingly, an inwardly rectifying current mediated by Kir2 channels in striatopallidal projection neurons is suppressed by acetylcholine through muscarinic receptor activation (Shen *et al.*, 2007). Thus, Kir2 channels are a major target for neuromodulation in the striatum.

The properties of the current suppressed by serotonin provide a simple explanation for the lack of effect of serotonin on FSI input resistance, despite the presence of robust depolarizations. Input resistance measurements were carried out at control resting membrane potential in both the absence and presence of serotonin (when FSIs were repolarized manually). In this voltage region (−73 to −61 mV), the slope conductance of the current suppressed by serotonin was extremely small in absolute value, i.e. < 0.3 nS. Classic studies on *N*-methyl-d-aspartate receptors have shown that the activation of a voltage-dependent current with negligible slope conductance in a certain voltage range results in no changes in the apparent cell input resistance measured in that region, whereas a paradoxical increase in input resistance is observed in a voltage region where the current has negative slope (Westbrook & Mayer, 1984; Mayer & Westbrook, 1985). In the present case, serotonin suppressed a voltage-dependent current; the magnitude of its slope conductance should be compared with the average FSI membrane conductance in control solution, which was *C* = 5.7 nS. The fractional contribution of the serotonin-suppressed conductance to the total conductance is Δ*C*/*C* ≤ (0.3 nS/5.7 nS) = 5.3%; the reciprocal relationship between resistance and conductance (*R* = 1/*C*) implies that Δ*R*/*R* = Δ*C*/*C*. Therefore, 5.3% is also an upper limit for the percent change in resistance caused by the serotonin-suppressed current. Whole-cell recordings are inevitably contaminated by thermal and synaptic noise, as well as by small variations in the electrode access resistance; it is therefore not surprising that such small changes in input resistance did not give rise to statistically significant effects. Consistently, 100 μm barium also did not induce significant changes in FSI input resistance, despite its action as an inward rectifier potassium current blocker. It should be noted, however, that suppression of an inwardly rectifying potassium current will still produce robust depolarizations and increases in excitability. These phenomena were indeed observed in our experiments. In a minority of serotonin-responsive FSIs, serotonin induced spontaneous firing, which was never present in these neurons in control solution; in the remaining ones, in the presence of serotonin, the ability of a depolarizing input to generate action potentials was strongly increased, even when the cells were repolarized to control levels. Furthermore, when stimuli that were suprathreshold in control solution were used, in the presence of serotonin, the latency of the first spike strongly decreased and the initial firing frequency strongly increased. This suggests that FSIs will be much more responsive to cortical glutamatergic inputs (Ramanathan *et al.*, 2002) in the presence of serotonin in the intact brain, resulting in stronger feed-forward inhibition of projection neurons. *In-vivo* studies have shown that FSIs respond earlier than projection neurons to cortical inputs (Mallet *et al.*, 2005). By exciting FSIs, serotonin may exacerbate this difference in latency, thus increasing the ability of FSI to limit the temporal extent of projection neuron responses and possibly suppressing responses altogether in those projection neurons that receive weaker cortical stimuli.

Approximately 22% of FSIs did not respond to the standard serotonin concentration used in this study (30 μm); the electrophysiological properties of these FSIs did not differ significantly from those of serotonin-responsive FSIs. In these cells, increasing the serotonin concentration up to 120 μm was also ineffective, suggesting that they belonged to a subpopulation that was unresponsive to serotonin. Previous histological studies have shown that the 5-HT_2C_ are not uniformly distributed in the striatum, being more densely expressed in the matrix or striosome compartments, respectively (Ward & Dorsa, 1996). Furthermore, the distribution of Kir2 channels is also uneven in the striatal compartments (Pruss *et al.*, 2003). It is possible that the expression of the 5-HT_2C_ receptors (or that of the potassium channels targeted by it) was low or absent in the FSIs that did not respond to serotonin, as a result of their compartmental location. Further studies will be required to clarify whether the FSIs that do not respond to serotonin belong to a specific striatal compartment.

The present observations on FSI extend our previous finding that serotonin excites striatal cholinergic interneurons (Blomeley & Bracci, 2005). It is worth noting that, although serotonin affects different membrane mechanisms in cholinergic interneurons and FSIs, in both cases the effects are mediated by 5-HT_2_ receptors. Cholinergic interneurons and FSIs control projection neurons in different ways but their actions on these cells may converge at a functional level. We have shown using paired recordings that cholinergic interneurons inhibit cortical glutamatergic input to projection neurons through a presynaptic mechanism (Pakhotin & Bracci, 2007). Projection neurons are also strongly affected by postsynaptic GABA_A_ receptors (Ade *et al.*, 2008) and are powerfully controlled by FSIs through activation of these receptors (Koos & Tepper, 1999; Tepper *et al.*, 2007). The present data, when paired with our previous observations on cholinergic interneurons, suggest that the presence of serotonin in the striatum will decrease the activity of striatal projection neurons through a combination of increased presynaptic inhibition of their glutamatergic input (caused by the excitation of cholinergic interneurons) and increased GABAergic postsynaptic inhibition (caused by the excitation of FSIs).

From a clinical perspective, it is interesting that selective serotonin reuptake inhibitors, which are widely used for the treatment of depression, can produce extrapyramidal motor effects, including akathisia and dystonia (Gerber & Lynd, 1998). The present results suggest that increased activity of striatal FSIs, due to prolonged activation of 5-HT_2_ receptors in patients exposed to selective serotonin reuptake inhibitors, may cause excessive suppression of the GABAergic striatal output and this could be an important causal factor in the genesis of motor side effects (Gubellini *et al.*, 2006).

## Abbreviations

5-HT: 5-hydroxytryptamine
FSI: fast-spiking interneuron
TTX: tetrodotoxin
